# Influence of PapMV nanoparticles on the kinetics of the antibody response to flu vaccine

**DOI:** 10.1186/s12951-016-0200-2

**Published:** 2016-06-10

**Authors:** Gervais Rioux, Damien Carignan, Alexis Russell, Marilène Bolduc, Marie-Ève Laliberté Gagné, Pierre Savard, Denis Leclerc

**Affiliations:** Department of Microbiology, Infectiology and Immunology, Infectious Disease Research Center, Laval University, 2705 Boul. Laurier, Quebec City, PQ G1V 4G2 Canada; Neurosciences, Laval University, 2705 Boul. Laurier, Quebec City, PQ G1V 4G2 Canada

**Keywords:** Adjuvants, Humoral response, PapMV nanoparticles, TLR7 agonist, Vaccine kinetics, Trivalent inactivated influenza vaccine, Influenza

## Abstract

**Background:**

The addition of an adjuvant to a vaccine is a promising approach to increasing strength and immunogenicity towards antigens. Despite the fact that adjuvants have been used in vaccines for decades, their mechanisms of action and their influence on the kinetics of the immune response are still not very well understood. The use of papaya mosaic virus (PapMV) nanoparticles-a novel TLR7 agonist-was recently shown to improve and broaden the immune response directed to trivalent inactivated flu vaccine (TIV) in mice and ferrets.

**Results:**

We investigated the capacity of PapMV nanoparticles to increase the speed of the immune response toward TIV. PapMV nanoparticles induced a faster and stronger humoral response to TIV that was measured as early as 5 days post-immunization. The addition of PapMV nanoparticles was shown to speed up the differentiation of B-cells into early plasma cells, and increased the growth of germinal centers in a CD4+ dependent manner. TIV vaccination with PapMV nanoparticles as an adjuvant protected mice against a lethal infection as early as 10 days post-immunization.

**Conclusion:**

In conclusion, PapMV nanoparticles are able to accelerate a broad humoral response to TIV. This property is of the utmost importance in the field of vaccination, especially in the case of pandemics, where populations need to be protected as soon as possible after vaccination.

**Electronic supplementary material:**

The online version of this article (doi:10.1186/s12951-016-0200-2) contains supplementary material, which is available to authorized users.

## Background

Vaccines are undeniably highly efficient in lowering the morbidity associated with many infectious diseases. However, it is also recognized that vaccines need to be improved. For example, the seasonal trivalent inactivated influenza vaccine (TIV) has an overall effectiveness of only 59 % in adults aged 18–65 years [1]. In elderly patients, vaccine efficiency is reduced, and new strategies are needed to enhance efficacy [2, 3]. Additionally, modern vaccine development is based mostly on recombinant protein technology, yielding highly purified molecules that are safer, but considerably less immunogenic, than live or attenuated pathogen vaccines [4, 5]. To compensate for this lack of antigen immunogenicity, the use of immune boosters or adjuvants in vaccine formulation has become a priority in today’s vaccine development.

Vaccine adjuvants are used primarily to prime a naive population; increase the duration of protection; drive the immune response toward a specific arm; increase the breadth of an immune response; and enhance the immune response in a immunodepressed population, all with minimal toxicity [6–8]. Activation of the innate immune system through engagement of toll-like receptors (TLRs), NOD-like receptors (NLRs), RIG-I-like receptors (RLRs), STING and C-type lectin receptor (CLRs) pathways to boost the adaptive immune response [4, 7, 9], are currently exploited for the discovery of new generation adjuvants. Surface (MPL and Flagellin) and intracellular [Imiquimod, CpG ODN, Poly(I:C)] TLR agonists, that trigger TLR4, 5, 7, 9 and 3, respectively, are potent inducers of the innate immune response, and have been shown to improve the resulting adaptive immune response against an antigen in a vaccination program [7, 10]. TLR7 and 8 have recently attracted attention because most human immune cells carry them; they are good inducers of interferon (IFN) type 1, and are the targets of choice for immunomodulatory molecules [11]. However, to date, no TLR7/8 agonist that can be used for prophylactic vaccination has been approved by regulatory bodies, mainly due to the toxicity seen with these agonists in clinical trials [12, 13]. There is a medical need for novel TLR7/8 agonists that can be used to improve the efficacy of prophylactic vaccines.

Papaya mosaic virus (PapMV) nanoparticles are composed of PapMV coat proteins assembled around a single-stranded RNA forming highly ordered rod-like structures of 100 nm long by 15 nm wide [14]. They can be utilised either as a vaccine platform [15–19], adjuvant [20, 21] or immunomodulator [22, 23], making them a particularly promising technology in the field of vaccination. In fact, PapMV nanoparticles improve the immune response toward TIV by enhancing the breadth and strength of the response 14 days after a single immunization [21]. In addition, PapMV can efficiently trigger innate immunity by stimulating secretion of proinflammatory cytokines and chemokines, and recruitment of immune cells in mice lungs [22]. Mechanistic studies have shown that PapMV nanoparticles can stimulate TLR7 [23], highlighting its role as a vehicle for the delivery of single-stranded RNA to immune cell endosomes. The Denis Leclerc y-4-14 14:12 induction of innate immunity through TLR7 in mice leads to secretion of IFN- and led to a significant decrease in tumour growth in the B16F10 model [24]. Although the adjuvant properties of PapMV nanoparticles with TIV have been investigated previously, its influence on the kinetics of the response to a vaccine has not yet been described thoroughly. The objective of this study was to determine the effect of PapMV nanoparticles on the kinetics and decision making pathways of the humoral immune response, using TIV as the model antigen.

## Results

**Fig. 1:**
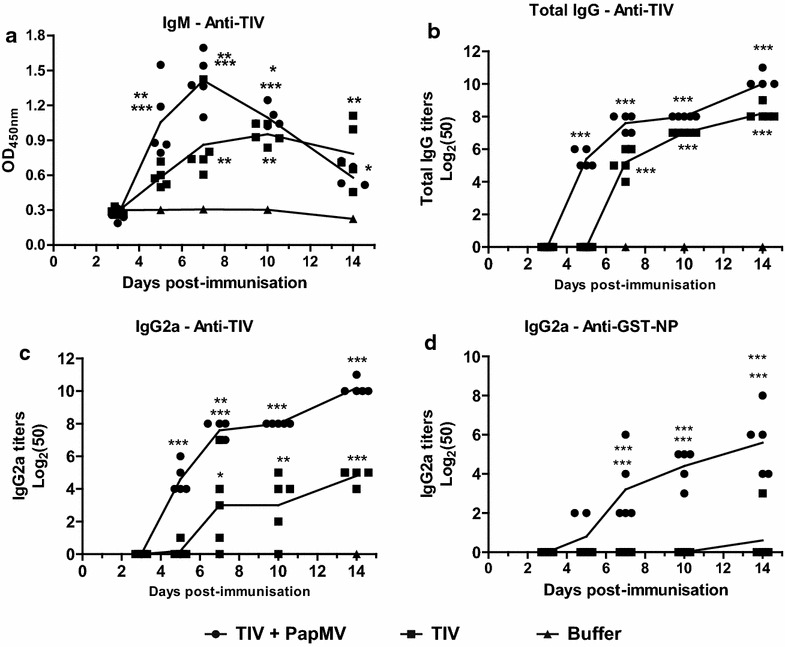
PapMV nanoparticles accelerate the humoral immune response against TIV. Blood samples from mice vaccinated with TIV adjuvanted with PapMV or TIV alone were collected at different time points (3, 5, 7, 10 and 14 days post-immunization) and assayed for IgM (**a**), total IgG (**b**) and IgG2a (**c**) against TIV and IgG2a against GST-NP (**d**) by enzyme-linked immunosorbent assay (ELISA). IgM titers are depicted as the O.D. using a serum dilution factor of 1:400. Data are shown as mean ± SEM, and significant differences are marked by *p < 0.05, **p < 0.01 and ***p < 0.001

PapMV nanoparticles accelerate the humoral immune response to TIV PapMV nanoparticles were recently shown to improve and broaden the immune response to TIV [21]. However, the speed at which PapMV nanoparticles induce an immune response against a vaccine has not yet been investigated. Therefore, we measured the antibody response in Balb/C mice immunized intramuscular (i.m.) once with either TIV alone (1 µg) or TIV (1 µg) adjuvanted with PapMV nanoparticles (15 µg) at different time points (3, 5, 7, 10 and 14 days post-immunization). Mice immunised with the adjuvanted vaccine exhibited significantly higher IgM titers against TIV starting at day 5 post- immunization as compared to mice immunised with TIV alone (Fig. 1a). This difference fades by day 10 as the antibody classes switch to IgGs (Fig. 1b, c). Consequently, mice immunised with the adjuvanted vaccine show accelerated production of TIV-specific IgG and IgG2a (Fig. 1b, c). Significant differences between titers in mice immunised with the adjuvanted or non-adjuvanted formulation are seen at all time points following the initial response observed at day 5. In a previous study, PapMV nanoparticles was shown to efficiently expand the breadth of a vaccine-elicited immune response by inducing a stronger response against non-immunogenic antigens, such as influenza nucleoprotein (NP) [20, 21]. Mice immunised with the adjuvanted vaccine exhibit faster and stronger NP-specific IgG2a production, starting at day 7 post-immunization (Fig. 1d). These results show that PapMV nanoparticles are able to induce a strong and broad humoral response toward vaccine antigens, as well as an immunoglobulin class-switch shortly after a single immunization.

### CD4+ T-cells are essential for the induction of a rapid immune response

**Fig. 2:**
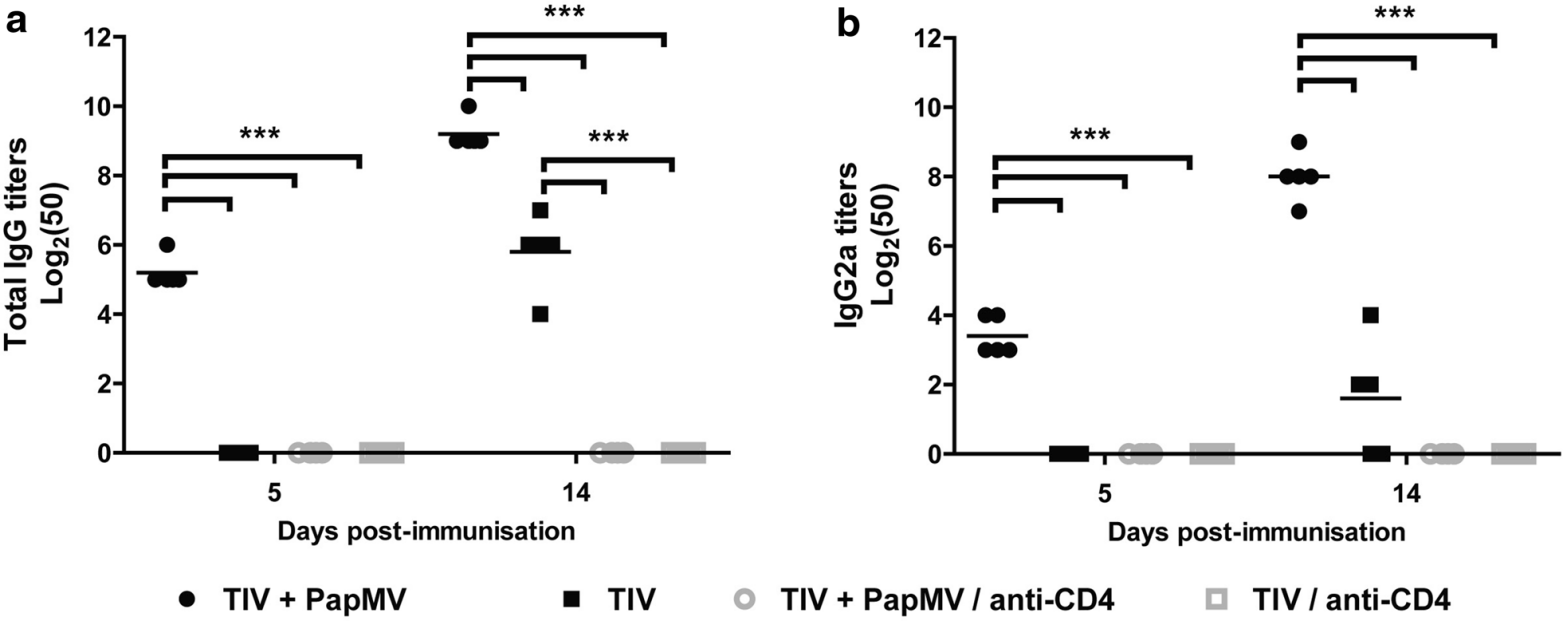
The early and late anti-TIV humoral response in PapMV nanoparticles adjuvanted vaccine is CD4-dependent. Blood samples from non-depleted (*black*, CD4+) or CD4-depleted (*gray*, CD4−) mice vaccinated with TIV containing PapMV nanoparticles or TIV alone were collected at 5 and 14 days post-immunization. Total IgG (**a**) and IgG2a (**b**) against TIV were assayed by ELISA. Data are shown as mean ± SEM, and significant differences are marked by ***p < 0.001

As asserted in other studies, TLRs agonists can overcome the dependency on the humoral response of CD4+ T-lymphocytes by directly activating B cells and rapidly inducing antibody production [25]. Thus, considering the immunomodulatory properties of PapMV nanoparticles, and the accelerated immune response induced by its addition to a vaccine, the dependency of CD4+ T cells was investigated. Mice were injected intraperitoneal (i.p.) with a CD4-specific antibody to deplete CD4+ T-cells. Depletion was confirmed by flow cytometry 24 h post-injection (Additional file 1: Figure S2). Depleted and non-depleted mice were immunised i.m. with either TIV alone (1 µg) or TIV adjuvanted with PapMV nanoparticles (15 µg) as before. The humoral response was measured at days 5 and 14 post-immunization. The depletion of CD4+ T cells completely abolished the initiation of a humoral response toward TIV in groups vaccinated with either TIV or TIV adjuvanted with PapMV nanoparticles (Fig. 2a, b). Those results show that the humoral response against TIV, regardless of the presence of PapMV nanoparticles, is dependent on CD4+ T cells. In the same manner, and since PapMV nanoparticles are proteinaceous in nature, we verified if a humoral response could be mounted toward PapMV nanoparticles in CD4-depleted mice. Antibodies against PapMV nanoparticles were also induced in CD4+ T cell-depleted mice, although the levels were lower than in undepleted mice (Additional file 2: Figure S3). PapMV nanoparticles are likely stimulating B cells and inducing a T-independent response against itself-a characteristic of highly ordered viral structures [26]. Interestingly, this property could not be transferred to TIV antigens when the adjuvanted formulation was used (Fig. 2).

### IgG-secreting plasma cell proliferation is enhanced by PapMV nanoparticles

**Fig. 3:**
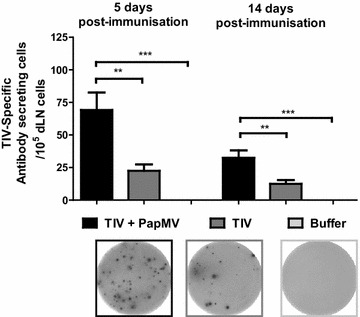
PapMV nanoparticles increase the number of TIV-specific plasma cells in an early response. TIV-specific IgG plasma cells were assayed by an enzyme-linked immunospot assay (ELISPOT) in the draining lymph nodes of vaccinated mice with TIV alone or together with PapMV nanoparticles. Data are shown as mean ± SEM, and significant differences are marked by **p < 0.01 and ***p < 0.001

The acceleration of antibody secretion must reflect a change in the population of B cells. Therefore, we evaluated the effect of PapMV nanoparticles on early and late differentiation of TIV-specific B cells into extrafollicular plasma cells in the proximal lymph nodes. We quantified the number of IgG-secreting cells specific for TIV in the draining (inguinal) lymph node early (5 days) and late (14 days) after a single immunization. As expected, PapMV nanoparticles were found to expand the number of TIV-specific plasma cells in the early and late stages of immune response development as compared to TIV alone (Fig. 3). The reduction in plasma cells at day 14 for both TIV and TIV adjuvanted with PapMV nanoparticles suggested that plasma cells entered apoptosis or migrated to other lymphoid tissues [27]. These results suggested that PapMV nanoparticles dictate the fate of B cells, directing increasing proliferation of early plasma cells and therefore the early production of antibodies.

### PapMV nanoparticles increase the size of germinal centers

**Fig. 4:**
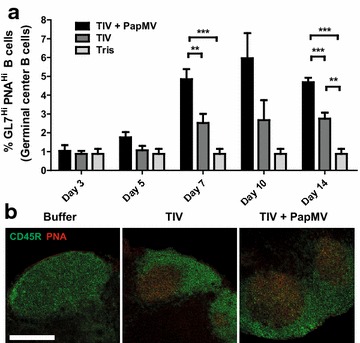
PapMV nanoparticles increase the proportion of germinal centers in the late response in TIV vaccinated mice, **a** Draining lymph nodes of mice immunised with TIV supplemented with PapMV or TIV alone were collected at different time points post-immunization (3, 5, 7, 10 and 14 days). Germinal centers (PNAhi and GL7hi) were assayed among CD45R+ cells by flow cytometry, **b** Spleen labelled with CD45R and PNA specific antibodies revealed that animals vaccinated with TIV + PapMV nanoparticles show larger germinal centers as compared to the control groups.

To determine the effect of PapMV nanoparticles on germinal centers (GC)-a key structure in the humoral affinity maturation [28]-we measured the number of GCs by flow cytometry by staining with PNA and for T- and B cell activation antigen (GL7) on CD45R-positive cells. The adjuvanted vaccine increased the magnitude of GC development in the draining (inguinal) lymph nodes starting at day 7 as compared to TIV alone (Fig. 4). In the adjuvanted formulation, the GC cells were twice as abundant than with TIV alone. This observation was confirmed in frozen sections of inguinal lymph nodes observed by confocal microscopy. As in the flow cytometry experiments, GCs were larger in mice immunised with the adjuvanted vaccine at day 14 (Additional file 3: Figure S4). PapMV nanoparticles are therefore able to increase the number of mature B cells in GCs.

### PapMV nanoparticles induce early and broad protection to a heterologous influenza strain

**Fig. 5:**
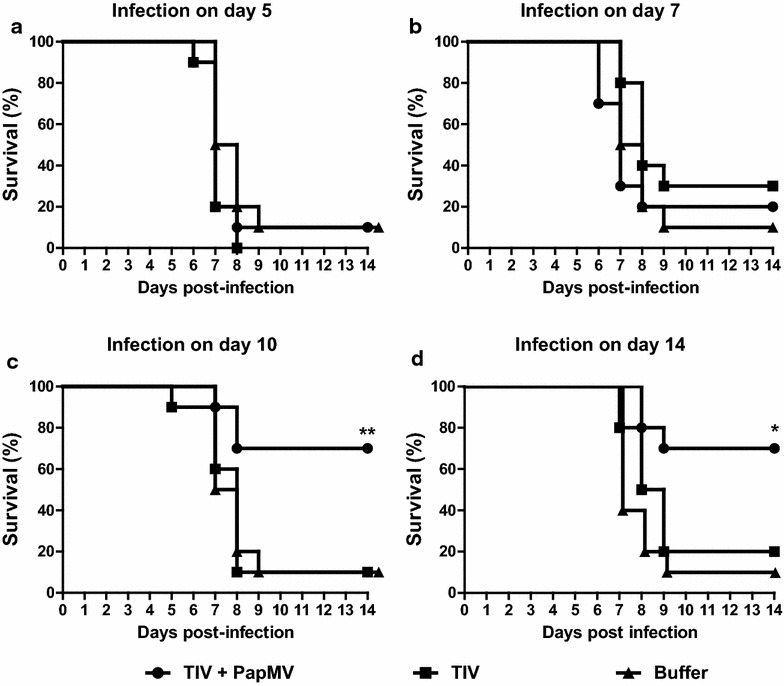
PapMV nanoparticles protect mice from an influenza virus challenge from day 10 post-immunization. Vaccinated mice were challenged with a lethal dose of influenza virus [A/WSN/33 (H1N1)] at different time points; 5 (**a**), 7 (**b**) 10 (**c**), (**d**) 14 days post-immunization. Mice were euthanized when their weight was equal to, or lower than, 20 % of their initial weight. Kaplan–Meier survival curves were analysed by the log rank test (**p < 0.01)

To determine if early influenza infection could be prevented by the use of PapMV with TIV, immunised mice were challenged with the heterologous influenza strain A/WSN/33 (H1N1) at early time points post-immunization (5, 7 and 10 days). This strain differs from those contained in TIV, to which it does not trigger a protective immune response. As expected, mice vaccinated with TIV alone were not protected from a lethal infection; meanwhile, mice immunized with the adjuvanted vaccine exhibited better protection against influenza virus infection as early as 10 days post-immunization (Fig. 5c). As expected, the same efficacy of protection was recorded at day 14 (Fig. 5d). We also showed they were less affected by the infection, as evidenced by lower weight loss in the vaccinated animals (Additional file 4: Figure S5) This result confirms that PapMV nanoparticles are able to induce a protective response against a lethal influenza virus challenge by improving a non-protective vaccine in a short time frame.

## Discussion

The results of this study have shown that PapMV nanoparticles speed up the kinetics of the antibody response toward TIV. This acceleration is dependent on CD4+ T lymphocytes; the proliferation and differentiation of B cells into antibody secreting cells is improved, and the size of the GCs early after immunization increases. The addition of the PapMV adjuvant also generates an immune response that allowed the animals to be rapidly protected against a heterologous influenza virus infection after a single immunization.

Although we could already detect antibodies against TIV as soon as 5 days post- immunization, following vaccination with PapMV nanoparticles, the protection against a lethal influenza challenge was found only in mice that received the vaccine 10 days before infection. Moreover, antibody titers against nucleoprotein (NP) reveal that the breadth of expansion of the humoral response takes longer than against more immunogenic antigens, such as HA and NA, contained in the vaccine. The addition of PapMV nanoparticles is nonetheless required to boost the response toward NP, and to protect mice against a lethal heterologous influenza virus challenge, as previously shown [21]. It is anticipated that the protection to influenza virus infection could be observed earlier if the infecting strain matched that used to make the vaccine, since antibody titers against other epitopes in the vaccine, e.g., HA or NA, seem to increase more rapidly.

PapMV nanoparticles were previously found to activate B and T lymphocytes, dendritic cells and macrophages 24 h after a single intravenous (i.v.) immunization, as revealed by the up-regulation of CD69, CD86 and MHC-I at the surface of immune cells [23]. This activation was abolished in TLR7, Myd88, Irf5/7 or Ifnar knockout mice, therefore highlighting the significant role played by those receptors, adapter proteins and transcription factors in the immune response induced by PapMV nanoparticles. The activation of the aforementioned cells is essential for the induction of a strong and sustained adaptive immune response against an antigen [29, 30]. This activation is also linked to B cell proliferation and differentiation into short-lived plasma cells, and in GC formation [31]. These parameters were all shown to be increased by PapMV in this study, and thus, in all likelihood, represent the outcome of the early innate activation that ultimately leads to a faster and more robust adaptive immune response.

Other adjuvants that trigger innate immunity by targeting TLR9 (CpG-ODN), and TLR7/8 [resiquimod (R848)], have been shown to increase the speed of antibody affinity maturation and GC development with an antibody or adjuvant conjugated model hapten, 4-hydroxy-3-nitrophenyl [32, 33]. The trigger of innate immunity through TLR agonists has been shown to increase antigen-specific and neutralizing antibodies. Consistent with this finding, persistance of GCs, and of plasma cell responses, which persisted in the lymph nodes to ensure a memory response, were also enhanced [34]. Since TLR agonists induce innate immunity and have been shown to be linked to an increased adaptive response in various studies [10], it is likely that other TLR agonists will also be able to increase the speed at which a humoral immune response is developed, but this still needs to be demonstrated when used with a vaccine formulation like TIV.

Finally, to further enhance the quality and the strength of the immune response to the flu antigen, it has been demonstrated that direct attachment to the TLR agonist can be of great benefit. For example, it was recently shown that linkage of the HA head to the phage Qb can enhance the immune response to the antigen, and trigger high levels of protection [35]. Therefore, since PapMV nanoparticles can also be used as a vaccine platform, it is tempting to speculate that direct attachment to nanoparticles could enhance the immune response directed to the antigen even further, as we have previously shown [36].

## Conclusion

In conclusion, PapMV nanoparticles-a TLR7 agonist-are able to accelerate the humoral response to a vaccine antigen. This feature is of major significance as it justifies the use of PapMV nanoparticles in vaccines where a rapid response against pandemic diseases is needed. To our knowledge, this is the first time that this property is described for an adjuvant in an actual commercial vaccine. This attribute is of the utmost importance, especially in case of pandemics where populations need to be protected as soon as possible after vaccination.

## Methods

### Adjuvants and antigens

PapMV nanoparticles (lot # Eng-1211) were provided by Folia Biotech Inc. (Quebec City, Quebec, Canada) and were produced as described in our previous study [21]. In brief, PapMV nanoparticles used in this report are self assembled in vitro with a non-coding ssRNA in vitro transcript of 1517 nucleotides. The nanoparticles show an average length of 100 nm and a diameter of 15 nm as shown by electron microscopy (Additional file 5: Figure S1A) and dynamic light scattering (Additional file 5: Figure S1B). The potential zeta was estimated at −6.12 mV (Additional file 5: Figure S1C), and is consistent with other batches of PapMV nanoparticles produced in the laboratory. LPS contamination was always below 50 endotoxin units (EU)/mg of protein, and was considered negligible.

The commercially available formulation of the trivalent inactivated influenza vaccine (TIV) 2013–2014 (lot #3YF27) (A/California/7/2009 NYMC X-179A (H1N1), A/Texas/50/2012 NYMC X-223A (H3N2) and B/Massachusetts/2/2012 NYMC BX-51B) from GlaxoSmithKline (GSK; Fluviral) was used as the model antigen.

### Mice immunization and depletion

The PapMV nanoparticles were formulated in 10 mM Tris pH 8.0 at a concentration of 0.5 mg/mL. TIV was prepared by GSK and contained 15 µg of each of the HA in a volume of 0.5 mL. Seven- to ten-week-old female BALB/c mice were immunised once by i.m. injection of 50 µL of a formulated vaccine, 1 µg (of each hemagglutinin) TIV+ 15 µg of PapMV nanoparticles, 1 µg (of each hemagglutinin) TIV or buffer (10 mM Tris pH8.0). Formulations were prepared and administered on the same day with syringes with 30G × 5/16? Needles. All the i.m. injection were in a final volume of 50 µL.

CD4 depletion was performed using 200 µg of rat anti-CD4 (Clone GK1.5) (Biolegend, San Diego, CA, USA) injected intraperitoneally (i.p.). Depleted mice were immunised 1 day post-depletion. Depleted mice were assayed for remaining CD4+ cells in the blood by flow cytometry, and immunised 1 day post-depletion. Cells were washed and stained with rat anti-CD4 (Clone GK1.5) (Biolegend, San Diego, CA, USA) and Alexa Fluor^®^ 488 goat anti-rat IgG (LifeTechnologies, Burlington, Ontario, Canada). Cells were analyzed by flow cytometry using a BD FACS Canto flow cytometer (BD, Mississauga, Ontario, Canada). The approval of this project for animal work is found under authorization number 2013-142 of the “Comité de Protection des Animaux-CHUQ (CPA-CHUQ)”.

### Antibody assay

Blood samples were collected from mice and centrifuged in BD microtainer blood collection tubes (BD, Mississauga, Ontario, Canada) for 2 min at 10,000*×g*. Considering ethical obligations and blood sampling limitations, each time point is represented by a different group of five mice. The serum were assayed for total IgG, IgG2a serotype, and IgM against TIV and IgG2a against GST-fused influenza nucleoprotein (GST-NP) by enzyme-linked immunosorbent assay (ELISA) as described elsewhere [15, 21]. IgGs ELISA were conducted by serial dilutions in twofold steps starting at 1 in 50 of serum in dilution buffer. Results are expressed as an antibody endpoint titer greater than threefold the background optical density values consisting of preimmune sera. IgM titers were measured by the optical density values of sera with a dilution factor 1:400.

### B cells enzyme-linked immunospot

Enzyme-linked immunospot (ELISPOT) 96-well PVDF-membrane microtiter plates (Millipore, Billerica, MA, USA) were activated with 50 µL of 35 % ethanol for 30 s, and washed five times with phosphate-buffered saline (PBS). Plates were then coated overnight with 2 µL of TIV in 100 µL PBS. Wells were then washed five times and blocked for 30 min with RPMI 1640 medium containing 10 % decomplemented foetal bovine serum. Mice lymph nodes were extracted at days 5 and 14 post-immunization and digested with type IV Collagenase (0.5 mg/mL) and DNase I (0.125 mg/mL) at 37 °C for 30 min. Digested lymph nodes were transferred into a 50 mL tube through a 70 µm mesh and washed with PBS +2 mM EDTA. Cells were then centrifuged at 500×*g* for 5 min at 4 °C and the supernatant was discarded. Lymph node cells (1 × 105) were deposited in the coated wells and incubated for 18 h at 37 °C, 5 % CO_2_. Antibodies were detected using a biotinylated anti-IgG (Life technologies, Burlington, Ontario, Canada) at 1 µg/mL and a streptavidin–alkaline phosphatase (Promega, Madison, WI, USA) diluted 1:1000. Spots were revealed with 100 µL of BCIP-NBT for 15 min, and then washed with distilled water. Plates were observed using a SMZ800 optical microscope (Nikon, Mississauga, Ontario, Canada) and counted automatically using ImageJ (Version 1.43 m, NIH, USA).

### Germinal center assay by flow cytometry

The lymph nodes (5 mice per group) were extracted at days 5 and 14 post- immunisation and digested with type IV Collagenase (0.5 mg/mL) and DNase I (0.125 mg/mL) at 37 °C for 30 min. Cells were stained with anti-mouse-CD45R-PerCP (0.2 µg, BD, Mississauga, Ontario, Canada), lectin PNA from *Arachis hypogaea*-AlexaFluor 647 (1 µg, Life technologies, Burlington, Ontario, Canada) and anti-mouse T- and B-cell activation antigen-FITC (Clone GL7) antibody (0.2 µg, BD, Mississauga, Ontario, Canada). Staining was done for 30 min at 4 °C and cells were then washed 3 times with 1 mL of cold flow cytometry buffer. Germinal centers were assayed on a flow cytometer by first gating for positive CD45R staining. Cells were considered to originate from GCs when they stained highly for PNA and GL7.

### Frozen section and confocal microscopy

Mice inguinal lymph nodes from the same side as the immunization site were extracted at day 14 and placed in RPMI 1640. Lymph nodes were then transferred into a mold containing premium frozen section compound (OCT) (VWR, Ville Mont-Royal, Quebec, Canada). Lymph nodes were then covered by OCT and snap-frozen in liquid nitrogen. 8-µm-thick sections were prepared using a Jung Cryostat 2800 Frigocut-E (Leica, Concord, Ontario, Canada) and applied to positively-charged slides. Sections were fixed for 20 min using 4 % paraformaldehyde in PBS and rinsed. Sections were then stained with anti-CD45R-AlexaFluor 488 (Clone RA3-6B2, Biolegend, San Diego, CA, USA) and PNA-AlexaFluor 647 (Life technologies, Burlington, Ontario, Canada) in staining buffer (Phosphate buffer + 5 % bovine serum albumin). Slides were mounted using SlowFade gold antifade mountant (Life technologies, Burlington, Ontario, Canada) and observed with a Quorum WaveFX (Quorum Technologies, Guelph, Ontario, Canada) confocal spinning disk microscope at 10×.

### Challenge with a heterologous influenza virus

Immunised mice, 10 per group, were challenged with 1.5 LD50 of A/WSN/33 (H1N1) influenza viruses at different time points post-immunization by an intranasal instillation of 50 µL viral preparation following anesthesia by isoflurane. Mice were monitored for 14 days for survival. Mice that lost 20 % or more of their initial weight were euthanized. Mice immunized with TIV and infected with A/WSN/33 (H1N1) develop viral symptoms and are not protected against a mild challenge. A/WSN/33 (H1N1) strain is therefore considered a heterologous strain in comparison to the strains contained in TIV.

### Statistical analysis of data

Data from more than two groups were analyzed with a parametric ANOVA test. Tukey’s post tests were used to compare differences among groups. Data from only two groups were compared using the *t* test. Kaplan–Meier survival curves were analysed by the log rank test. Values of *p < 0.05, **p < 0.01 and ***p < 0.001 were considered statistically nificant. Statistical analyses were performed with GraphPad PRISM 5.01 (GraphPad Software, La Jolla, California, USA).

## Additional files

10.1186/s12951-016-0200-2 Depletion of CD4 T-lymphocyte by intraperitoneal injection of specific CD4 antibodies. Mice were depleted of CD4 T-cells by an intraperitoneal injection of 200 µg of CD4-specific antibodies. Flow cytometry was conducted on mice blood samples collected 24 hours after injection.
10.1186/s12951-016-0200-2 PapMV nanoparticles induce a CD4-independent response against itself. Blood samples from non-depleted (black, CD4+) or CD4-depleted (gray, CD4-) mice vaccinated with TIV containing PapMV nanoparticles were collected at 5 and 14 days post-immunization. Total IgG (A) and IgG2a (B) against PapMV were assayed by ELISA. Data are shown as means ± SEM, and significant differences are marked by (***) p<0.001.
10.1186/s12951-016-0200-2 PapMV nanoparticles increase the size of germinal centers in the late response in TIV vaccinated mice. Draining lymph nodes of mice immunised with TIV supplemented with PapMV or TIV alone were collected at day 14 and germinal centers were snap-frozen, sectioned and stained (CD45R+ and PNAhi).
10.1186/s12951-016-0200-2 Weight losses of mice during the influenza virus infection. The weight losses of mice infected with influenza virus of Figure 5 was followed during 14 days post-challenge. Mice were euthanized when their weight was equal or lower than 20% of their initial weight.
10.1186/s12951-016-0200-2 Biochemical characterization of PapMV nanoparticles. Electron micrographs of PapMV nanoparticles (A) that harbour a rod-shape of 100nm as shown by dynamic light scattering (DLS) (B) . PapMV nanoparticles were in 10mM Tris buffer pH8.0.

## Abbreviations

CLR: C-type lectin receptor
GC: germinal centers
i.m.: intramuscular
i.p.: intraperitoneal
i.v.: intravenous
NLR: NOD-like receptor
NP: nucleoprotein
PapMV: papaya mosaic virus
RLR: RIG-I-like receptor
TIV: trivalent inactivated influenza vaccine
TLR: toll-like receptor
