# Supplementary figures and images for: Influence of PapMV nanoparticles on the kinetics of the antibody response to flu vaccine
Source: J Nanobiotechnology. 2016 Jun 10;14:43. doi: 10.1186/s12951-016-0200-2 (PMC4901503; doi:10.1186/s12951-016-0200-2)

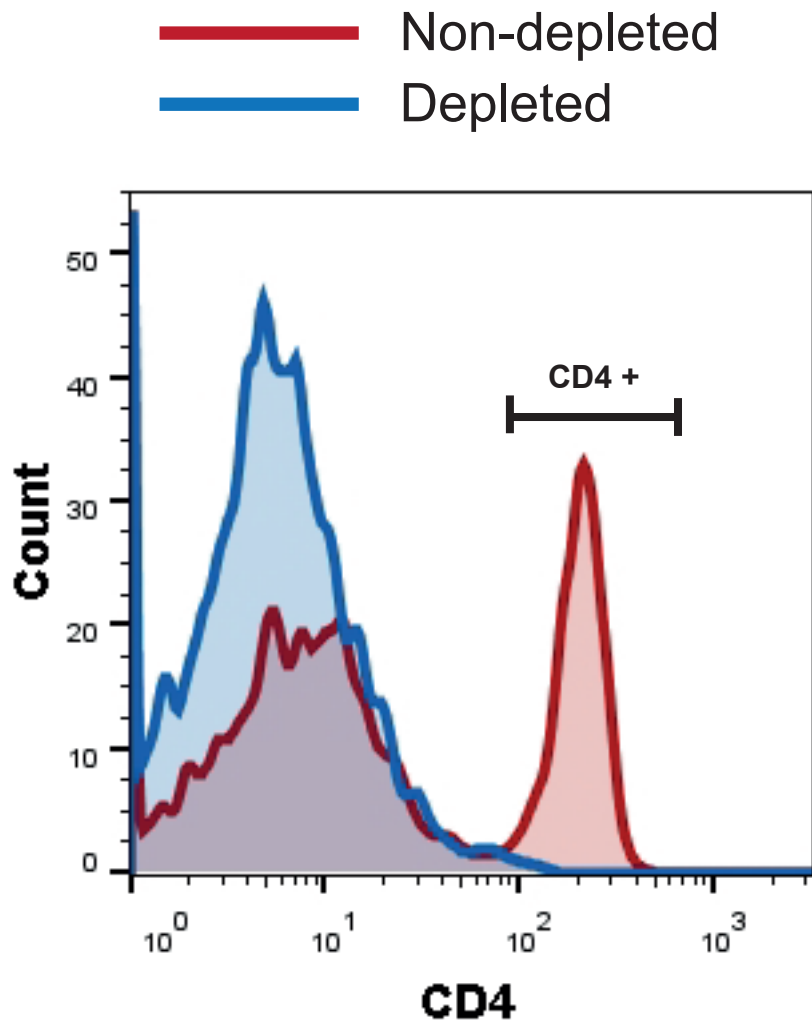

Supplement: Supplementary file 1 — 10.1186/s12951-016-0200-2 Depletion of CD4 T-lymphocyte by intraperitoneal injection of specific CD4 antibodies. Mice were depleted of CD4 T-cells by an intraperitoneal injection of 200 µg of CD4-specific antibodies. Flow cytometry was conducted on mice blood samples collected 24 hours after injection. [file 12951_2016_200_MOESM1_ESM.pdf]

**A**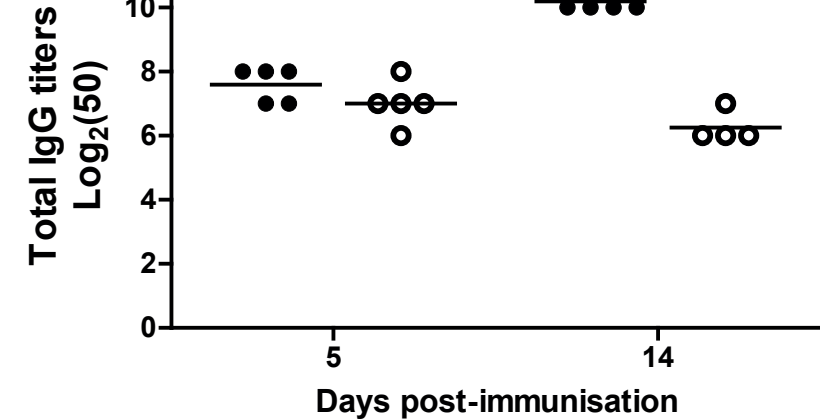

● TIV + PapMV

**B**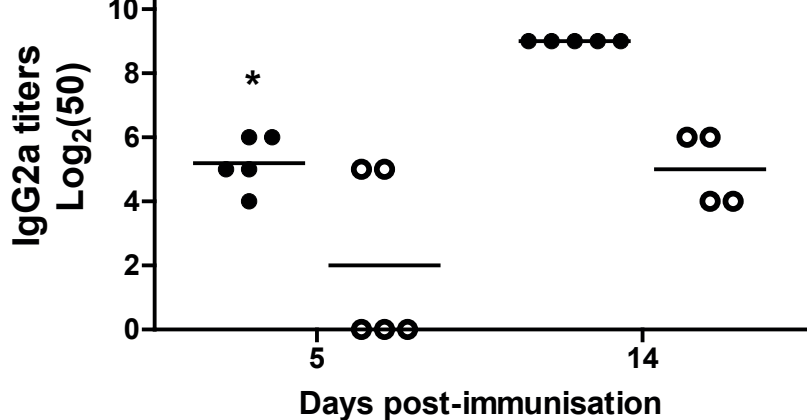

○ TIV + PapMV - anti-CD4

Supplement: Supplementary file 2 — 10.1186/s12951-016-0200-2 PapMV nanoparticles induce a CD4-independent response against itself. Blood samples from non-depleted (black, CD4+) or CD4-depleted (gray, CD4-) mice vaccinated with TIV containing PapMV nanoparticles were collected at 5 and 14 days post-immunization. Total IgG (A) and IgG2a (B) against PapMV were assayed by ELISA. Data are shown as means ± SEM, and significant differences are marked by (***) p<0.001. [file 12951_2016_200_MOESM2_ESM.pdf]

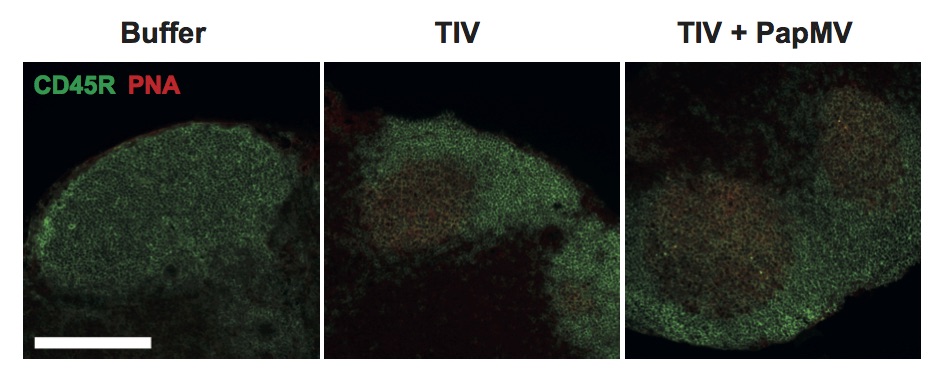

Supplement: Supplementary file 3 — 10.1186/s12951-016-0200-2 PapMV nanoparticles increase the size of germinal centers in the late response in TIV vaccinated mice. Draining lymph nodes of mice immunised with TIV supplemented with PapMV or TIV alone were collected at day 14 and germinal centers were snap-frozen, sectioned and stained (CD45R+ and PNAhi). [file 12951_2016_200_MOESM3_ESM.jpg]

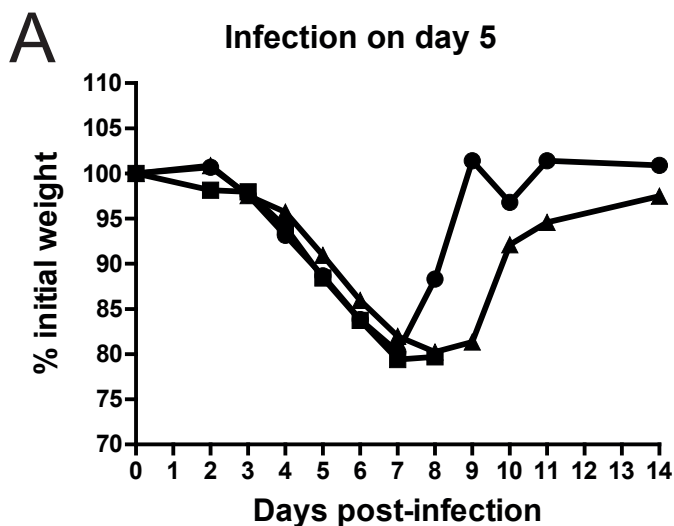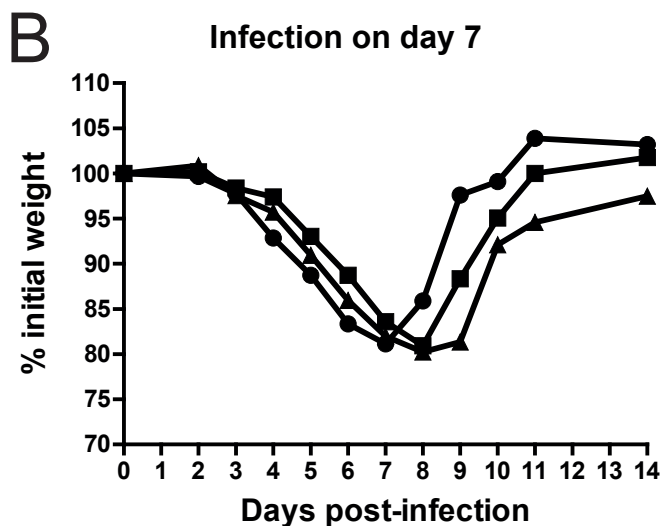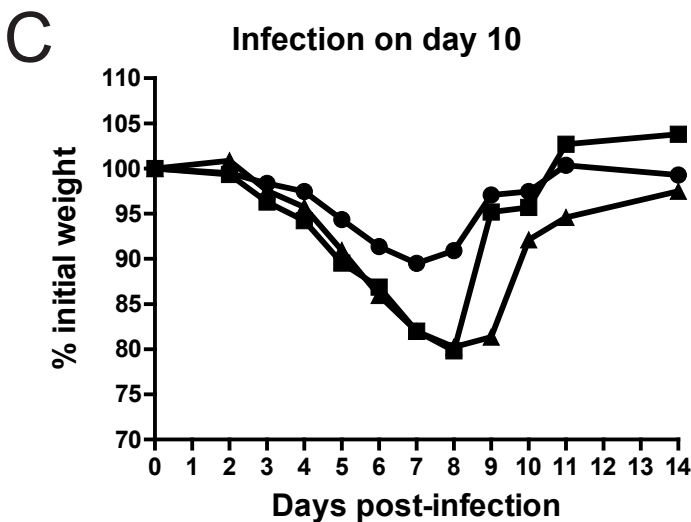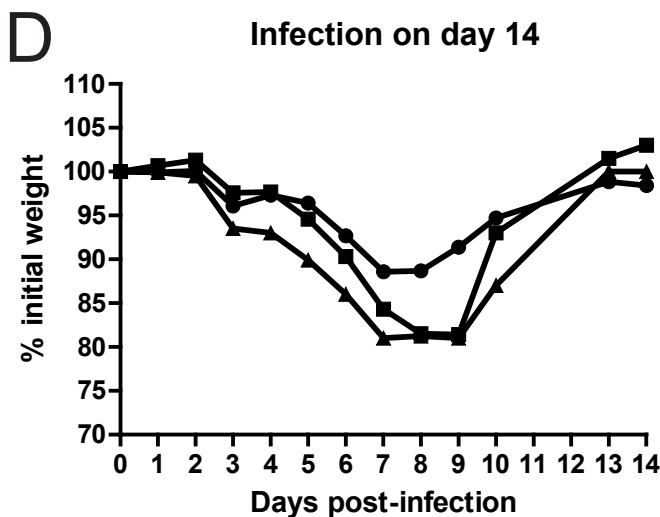

● TIV + PapMV

■ TIV

▲ Buffer

Supplement: Supplementary file 4 — 10.1186/s12951-016-0200-2 Weight losses of mice during the influenza virus infection. The weight losses of mice infected with influenza virus of Figure 5 was followed during 14 days post-challenge. Mice were euthanized when their weight was equal or lower than 20% of their initial weight. [file 12951_2016_200_MOESM4_ESM.pdf]

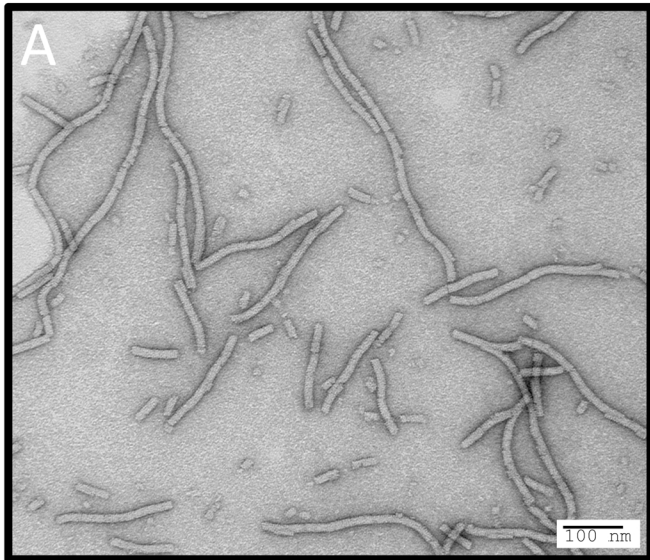

**B** 4 measurements:

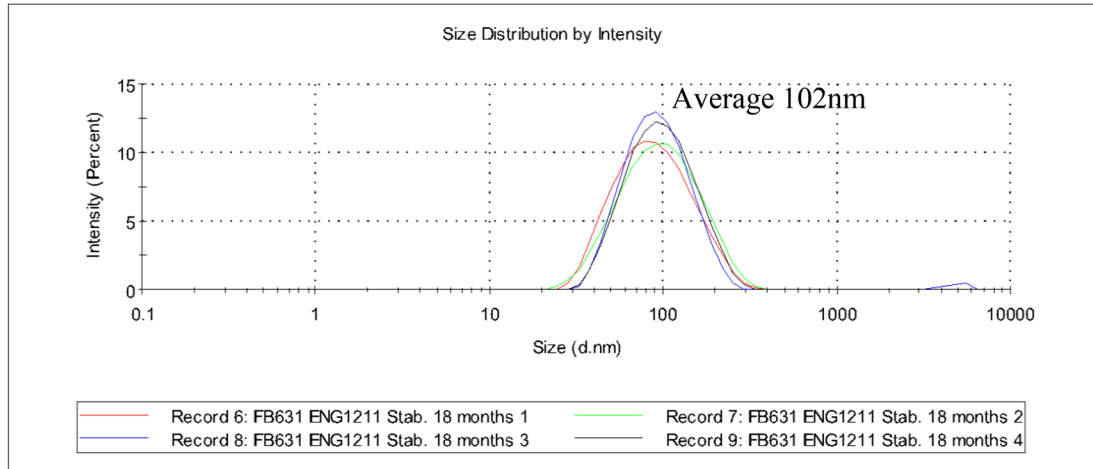

Supplement: Supplementary file 5 — 10.1186/s12951-016-0200-2 Biochemical characterization of PapMV nanoparticles. Electron micrographs of PapMV nanoparticles (A) that harbour a rod-shape of 100nm as shown by dynamic light scattering (DLS) (B) . PapMV nanoparticles were in 10mM Tris buffer pH8.0. [file 12951_2016_200_MOESM5_ESM.pdf]
